# Rural Zulu women’s knowledge of and attitudes towards Pap smears and adherence to cervical screening

**DOI:** 10.4102/phcfm.v11i1.1994

**Published:** 2019-10-03

**Authors:** Michelle A.L. Godfrey, Sithokozile Mathenjwa, Nasim Mayat

**Affiliations:** 1 Lower Umfolozi District War Memorial Hospital, Empangeni, KwaZulu-Natal, South Africa; 2 Department of Gynaecological Oncology, Queen Alexandra Hospital, Portsmouth, United Kingdom

**Keywords:** cervical pap smear, cervical screening, human immunodeficiency virus, cervical cancer, human papillomavirus, Zulu women, South Africa

## Abstract

**Background:**

Cervical cancer is the most common form of cancer in black women in South Africa and has almost a 60% mortality rate. However, adherence to cervical screening programmes of black women living in rural South Africa is not universal.

**Aim:**

The aim of this study was to gain a better understanding of rural Zulu women’s knowledge of and attitudes towards Pap smear tests, and their reasons for participation or non-compliance with cervical screening.

**Setting:**

This study was conducted at the gynaecology and antenatal clinics in a secondary referral hospital in rural KwaZulu-Natal.

**Methods:**

A hospital-based cross-sectional study was undertaken in the form of a semi-structured patient questionnaire survey with open and closed questions. The responses to the open-ended questions were manually analysed by the authors using a thematic approach. Outcome measures included whether the woman had a previous Pap smear, her understanding of the cervical screening programme and causes of cervical cancer.

**Results:**

This study included a total analysis of 234 responses. The mean age was 29 years (s.d. = 8.3 years). Overall, 32.5% of women had previously had a Pap smear. Among the responders, 33.3% were human immunodeficiency virus (HIV)-positive and 53.0% were HIV-negative. Only 19.2% of women understood that a Pap smear was related to screening for cervical cancer.

**Conclusions:**

This study illustrated a poor understanding of cervical screening, which may result in the low level of uptake of Pap smear reported; this is particularly concerning in HIV-positive women, who are at higher risk of developing cervical cancer. Urgent and extensive public health campaigning is required within rural South Africa to improve cervical screening uptake and decrease cervical cancer mortality.

## Introduction

Cervical cancer is the second most common cancer in women across the world, with approximately 85% occurrence rate in the developing world.^1^ It is the leading cancer among black women in South Africa.^2^ The cervical cancer crude incidence rate is 44.4 per 100 000 women per year in South Africa.^3^ Cervical cancer is the leading cause of female deaths in women aged 15–44 years in South Africa, with approximately a 60% mortality rate.^4^ This is despite cervical cancer stating a recognised treatable premalignant condition that can be detected during a Pap smear test.

Cervical screening has significantly lowered cervical cancer mortality in developed countries, with a reduction in 49% of cases in the United Kingdom following the introduction of a national screening programme.^5^ It has been found that having just one Pap smear reduces the risk of cervical cancer significantly.^6^ In rural South Africa, it has been reported that only 18% of women had previously had a Pap smear.^7^ Cervical cancer is predominantly caused by human papillomavirus (HPV), an easily acquired sexually transmitted infection. A vaccine is now available to protect young girls against risk of acquiring HPV and is offered before the girl becomes sexually active.^8^ However, there are generations of women who have already been exposed to HPV, before the development of the vaccine. For these women, cervical screening is essential to pick up premalignant cervical changes and implement treatment before cervical cancer develops.

South Africa has a free national screening programme involving three Pap smears in a lifetime, at 10-yearly intervals from age 30 years for low-risk human immunodeficiency virus (HIV)-negative women.^8^ The World Health Organisation (WHO) recommends 3-yearly Pap smears in HIV-positive women.^9^ The South African national cervical screening policy^8^ was updated in 2017 that is in line with the WHO recommendations, advising all HIV-positive women should be screened at the time of their HIV diagnosis and then every 3 years if cytology is negative, and yearly if cytology shows any abnormality. However, this policy is yet to be widely implemented. Women with HIV are approximately 5 times more likely to develop premalignant cervical changes because of immunosuppression and the high prevalence of concurrent HPV infection.^10^ An estimated 3.4 million women are living with HIV or AIDS (acquired immunodeficiency syndrome) in South Africa. The incidence of HIV among women attending antenatal clinics in 2013 in KwaZulu-Natal was 40.1%,^11^ increased from 37.5% in 2011.^12^ A South African study found HIV-positive women presented up to 18 years earlier with cervical cancer than HIV-negative women.^13^

This study aimed to gain a better understanding of a rural population of predominately Zulu women’s knowledge of and attitudes towards Pap smear tests, and their reasons for participation or non-compliance with cervical screening. There is little existing information in the literature regarding rural Zulu women’s health beliefs towards cervical screening and exploration of this subject may help to enhance patient-led educational programmes to improve cervical screening uptake and decrease cervical cancer mortality.

## Methods

### Study design and analysis

An exploratory hospital-based cross-sectional study was undertaken, in the form of a semi-structured patient questionnaire survey with open and closed questions. The survey was devised with hospital staff input, including Zulu staff members, and translation into the local language isiZulu to ensure comprehension and cultural sensitivity. Answers in isiZulu were translated into English by a native Zulu speaker who was university educated and fluent in the English language in the process of reverse translation. All surveys were translated by the same individual to ensure consistency.

The survey involved 18 questions. Fourteen questions were closed-ended, in a tick box format, with additional space for comments. These included age, parity, education status, contraception use, HIV status, previous Pap smear history and result. Data from the closed-ended questions were entered in Excel and descriptive statistics were used to analyse the results.

Four open-ended questions involved determining why the women had or had not previously had a Pap smear:

- Why did you have a Pap smear?
- Why have you never had a Pap smear?
- What is a Pap smear for?
- What causes cervical cancer?

The responses to the open-ended questions were manually analysed by the authors using a thematic approach. Similar written responses were grouped into critical categories by the authors, as there were several recurring themes and answers to the open questions. For example, written answers for causes of cervical cancer that included multiple sexual partners, early sexual debut and sexually transmitted infections were grouped as a category and expressed in descriptive statistics. The codes were compared and modified until a consensus was reached among the authors. No answers have been discredited or omitted to prevent author bias. We have also included some direct written statements used by the study population, rather than attempt to translate into medical terminology to avoid bias. For example, some respondents believe that cervical cancer is caused by ‘an unclean womb’. This is the closest English translation from the Zulu phrase, and we have directly quoted the respondents to gain maximum insight into the population’s health beliefs regarding Pap smears and cervical cancer.

### Setting

The study site was a secondary referral obstetrics and gynaecology hospital, Lower Umfolozi District War Memorial Hospital, in rural KwaZulu-Natal. The hospital received referrals from 16 level 1 hospitals across a large area of rural northern KwaZulu-Natal. The study continued for 2 weeks in March 2013.

### Study population

The population was predominately isiZulu-speaking South African women attending the antenatal or gynaecology clinics. The women attending the clinics had complex problems, with a prevalence of HIV of 40.1% of women attending antenatal clinics in KwaZulu-Natal in 2013,^11^ and come from several remote communities. There is little published data on Zulu women’s knowledge of and attitudes towards Pap smears; therefore, we consider this an exploratory study. An average of 1000 women attend hospital outpatient clinics per month. We aimed to sample 30% of the study population, in keeping with other exploratory studies.^14^ A sample size of 244 women represents 30% of the attendees per month, with a 95% confidence interval and 5% error margin. A 30% sample size was determined feasible to achieve given the staff work pressures and the resources available while maintaining an adequate response rate to represent the population.

A random sample of women attending antenatal and gynaecology appointments was invited to participate in the survey. The nurse in the clinic explained the purpose of the study and women were invited to join. The information sheet, consent form and questionnaire were available in isiZulu and English. The nurse was not a member of the research team.

Out of 280 questionnaires distributed, a total of 247 women voluntarily filled out the survey. The women were invited to fill out the survey while they were waiting for their appointments. If the patient was unable to read or write, then the clinic nurses interviewed the patients in private rooms and documented the answers.

### Ethical considerations

As per local guidelines, ethical approval was granted by the study site’s ethics committee. Informed consent of participants was obtained. No identifiable patient information was included in the survey. Women under the age of 18 years were excluded. No incentive was provided and participation or declining involvement did not affect their treatment at the hospital.

## Results

There were 247 gathered responses and 234 met the inclusion criteria and analysed. Thirteen women were excluded for being under the age of 18 years (*n* = 8) or not entering their age (*n* = 5). The demographics of the cohort are shown in Table 1. The mean age was 29 years (standard deviation [s.d.] = 8.3 years, range 18–70 years) and 34.6% (*n* = 81) women were aged 30 and above. Ninety-five per cent (*n* = 223) women were black and answered in isiZulu. Overall, 66.7% of women had completed education grade 10 and 9.0% (*n* = 21) had a university education. One-third of women (*n* = 79) were HIV-positive. Of the HIV-positive women, 93.7% (*n* = 74) entered their year of diagnosis. Early acquisition of HIV as a teenager had affected 6.8% (*n* = 5) of women and 5.4% (*n* = 4) had HIV for over 10 years. Data on their latest cluster of differentiation 4 (CD4) count were supplied by 81.0% (*n* = 64) of the HIV-positive women, with 10 women reporting a CD4 count of less than 200 (Table 1).

**TABLE 1:** Population demographics.

Demographic	%	*n*
**Age (years)**		
18–30	65.4	153
31–40	27.8	65
41–50	3.8	9
51–60	1.7	4
61–70	1.3	3
**Ethnicity**		
Black people	95.3	223
Caucasian	1.7	4
Mixed race	1.3	3
Did not answer	1.7	4
**Education**		
Grades 2–9	17.9	42
Grades 10–12	66.7	156
University	9.0	21
Did not answer	6.4	15
**Parity**		
0	5.6	13
1	36.8	86
2	28.6	67
3	12.0	28
4 and over	12.4	29
Did not answer	4.7	11
**Contraception**		
None	41.0	96
Depo-Provera	26.9	63
Oral contraceptive pill	8.5	20
Intrauterine device	2.1	5
Sterilised	1.7	4
Condoms	0.9	2
Did not answer	18.8	44
**Marital status**		
Single	2.6	6
Partner	59.4	139
Married	25.2	59
Widow	1.7	4
Did not answer	10.7	25
**HIV status**		
Positive	33.3	33.3
Negative	53.0	53.0
Do not know	3.8	3.8
Declined to answer	9.0	9.0
**Years since HIV diagnosis**		
0–4 years	62.0	49/79
5–9 years	26.6	21/79
10 years and over	5.1	4/79
Did not answer	6.3	5/79
**CD4 count**		
Less than 200	12.8	10/79
200–400	29.1	23/79
401–600	21.5	17/79
Over 600	17.7	14/79
Did not answer	19.0	15/79

In total, 32.5% (*n* = 76) of women had previously had a Pap smear (Table 2). For women aged 30 and above, this percentage increased to 48.4% (*n* = 46). In the group of HIV-positive women, 39.2% (*n* = 31) had had a Pap smear. Overall, 58.5% (*n* = 137) of the women were eligible for a free Pap smear because of being age 30 and above or being HIV-positive, and in this cohort, 42.3% (*n* = 58) had received a Pap smear. Of the HIV-positive women aged less than 30 years, 28.6% (*n* = 12) had previously had a Pap smear.

**TABLE 2:** Pap smear history.

Question	%	*n*
**Have you ever had a Pap smear?**		
Yes	32.5	76
No	66.2	155
Did not answer	1.3	3
**Number of Pap smear**		
1	57.6	44/76
2	19.7	15/76
3	5.3	4/76
4+	5.3	4/76
Did not answer	11.8	9/76
**What was the result?**		
Normal	71.1	54/76
Colposcopy needed	3.9	3/76
Don’t know the result	10.5	8/76
Awaiting results	7.9	6/76
Did not answer	6.6	5/76

Reasons for having a Pap smear were varied (Table 3). Most women had received a Pap smear as part of an investigation for symptoms (57.9%; *n* = 44), not as an asymptomatic screening. An additional 42.1% (*n* = 32) had a Pap smear because the clinic had offered it to them. The most commonly cited reason for not having a Pap smear was ‘never heard of a Pap smear’ (27.1%; *n* = 42). One in five (19.4%; *n* = 30) women felt ‘too scared’ to have one. Overall, 12.9% (*n* = 20) stated the reason as ‘never offered one’. Other reasons included ‘feeling well, so Pap smear not needed’ (5.8%; *n* = 9), not knowing when/where to have one (6.5%; *n* = 10) and not wanting to know the result (1.9%/*n* = 3).

**TABLE 3:** Open-ended questions exploring the women’s understanding of Pap smears and cervical cancer.

Open-ended questions	%	*n*
**Why did you have a smear test?**		
Offered to them by clinic	42.1	32/76
Abnormal vaginal discharge	30.3	23/76
Abdominal pain	14.5	11/76
Post-coital/abnormal bleeding	9.2	7/76
Fibroids	2.6	2/76
Genital warts	1.3	1/76
**Why have you never had a smear test?**		
Never heard of smear test	27.1	42/155
Too scared/too painful	19.4	30/155
Never offered one	12.9	20/155
Feel well so don’t need one	5.8	9/155
No time to get one/too lazy	5.8	9/155
Did not know where or when to have one	6.5	10/155
Do not want to know result	1.9	3/155
Not old enough	1.9	3/155
Did not answer	18.7	29/155
**What is a smear test for?**		
Check health status	22.2	52
Detection of cancer	19.2	45
Check for disease of womb	3.8	9
To check for childbirth related problems	3.0	7
I don’t know	2.1	5
Did not answer	49.6	116
**What causes cervical cancer?**		
Don’t know	16.2	38
Multiple sexual partner/early debut/STI	14.1	33
‘Unclean womb’	2.1	5
Prolonged bleeding	1.3	3
‘Eating soil’	1.3	3
Smoking	1.3	3
Hereditary factor	0.9	2
Drugs	0.9	2
‘Keeping a cell phone in bra’	0.9	2
Did not answer	61.1	143
**Is there any treatment for cervical cancer?**		
Yes	27.8	65
No	10.3	24
Don’t know	50.0	117
Did not answer	12.0	28

In response to ‘What is a Pap smear for?’, almost one in five women (19.2%; *n* = 45) stated that it was for the detection of cancer and 22.2% (*n* = 52) of women responded that it was to ‘check your health status and get treatment if needed’.

The question ‘What causes cervical cancer’ had the lowest response rate, with 61.1% (*n* = 143) leaving the answer blank and 16.2% (*n* = 38) writing ‘I don’t know’. Overall, 14.1% (*n* = 33) mentioned sexually transmitted diseases/multiple sexual partners/early sexual debut. Other answers included ‘an unclean womb’ (2.1%; *n* = 5), smoking (1.3%; *n* = 3), prolonged bleeding (1.3%; *n* = 3), ‘eating soil’ (1.3%; *n* = 3), ‘keeping cell phones in your bra’ (0.9%; *n* = 2), using drugs (0.9%; *n* = 2) and hereditary influence (0.9%; *n* = 2).

## Discussion

### Main findings

Overall, 42.3% of women meeting the WHO criteria^8^ had previously had a Pap smear. Only 39.2% of the HIV-positive women had had a Pap smear. There was a particularly low level of Pap smear participation in the HIV-positive women aged less than 30 years (28.6%).

Human immunodeficiency virus-positive women develop cervical cancer much earlier than HIV-negative women, and we can extrapolate that those with early acquisition of HIV are at risk of developing cervical cancer before the usual age of screening.^10,13^ Many countries recommend an annual Pap smear for HIV-positive women.^15^ A South African study found HIV-positive women presented with cervical cancer up to 18 years earlier than HIV-negative women, with approximately 16% of invasive cervical cancers occurred before the age of 30 years in the HIV-positive cohort.^13^ Therefore, the early premalignant changes would have most likely developed during their 20s. Most women knew their HIV status; however, from our study, it appears the need for additional Pap smears has not been widely promoted.

Over half (57.9%; *n* = 44) of the Pap smears were used to investigate abnormal symptoms, demonstrating a low level of provision of an asymptomatic cervical screening Pap smear. In addition, over one in four women had not had a Pap smear because they ‘do not know what a Pap smear is’, while 5.8% felt it was an unnecessary test if they did not have symptoms. Several of these findings are amenable to patient education. One in five women reported that they had not had a Pap smear as they ‘felt scared’, in keeping with a previously reported South African study.^7^

A positive finding is that 42.1% of women had a Pap smear because it was offered to them by the clinic, showing cultural acceptance of the test and a willingness of the women to check for diseases. It is also positive that despite many women not knowing what a Pap smear is for, many answered that they ‘would like to know more about it’ through our survey. It is evident from our study that the women had a low level of fatalism and are keen to look after their health, with only 1.9% not having a Pap smear because of not wanting to know the result.

It also demonstrates that opportunistic screening may be one way of increasing rates of uptake. Research has shown that offering screening in the workplace can effectively increase cervical Pap smear uptake rates in middle-income countries.^16^

There was a low level of knowledge for what causes cervical cancer. Only 14.1% stated sexually transmitted diseases/early sexual debut and multiple sexual partners. Poor understanding of the disease is in accordance with other evidence from Southern Africa.^17^ A study in Gaborone showed that approximately half of the respondents said they did not know the causes of cervical cancer.^17^ There were other interesting beliefs reported – such as ‘eating soil’ and ‘drugs’, ‘having an unclean womb’ and ‘keeping a cell phone in bra’ – that women thought the causes of cervical cancer. This information is helpful for health professionals to address and educate the local population.

## Strengths and limitations

The views and opinions of Zulu women in rural areas are under-represented in the literature regarding reasons for poor Pap smear uptake. This study is a prospectively gathered questionnaire asked in the local language, which has provided valuable insights into health misconceptions and opportunities to instigate patient education to improve attendance for Pap smear screening. The component of open-ended questions allowed the women to expand on their answers, aiding identification of incorrect health beliefs.

The short study period of 2 weeks aimed to ensure that the same women were not asked to fill out the survey multiple times while meeting the required calculated sample size. However, a more extended study period may have provided additional unique data on the views of Zulu women. Also, further scrutiny of some answers like ‘I don’t know what causes cervical cancer’ hides less socially accepted beliefs. There may be other attitudes not disclosed within this written survey, which may be revealed via other means, such as face-to-face interviews. Some women may have the correct knowledge regarding Pap smears, but may not participate in screening because of concealed reason, which requires further study and consideration.

The women were attending antenatal or gynaecological clinics, indicating that they are already engaged in healthcare and may have some knowledge of gynaecological health problems. The study site is a regional obstetrics and gynaecological secondary referral centre. It is also in an urbanised area, although it receives referrals from a large geographic area of mostly rural communities. However, women engaged in travelling to the hospital may have a higher level of understanding than their community peers. A more isolated Zulu population of KwaZulu-Natal may have less engagement, access and knowledge of Pap smears.

Future studies of interest would involve determinants of Pap smear participation in the HIV-positive women, as only 39.2% of HIV-positive women had ever had a Pap smear in our study, and this falls to 28.6% for HIV-positive women under the age of 30 years. Human immunodeficiency virus-positive women are a key target population to improve Pap smear adherence as they are at higher risk of developing cervical cancer, compared with HIV-negative women.

## Conclusion

Awareness through education is an important way to empower women to participate in cervical cancer screening programmes actively. It is particularly crucial that HIV-positive women, who usually have regular contact with community health service, should be offered and encouraged to have cervical screening. Urgent and extensive public health campaigns are required in rural South Africa to educate women about the potential lifesaving benefits of having a regular Pap smear to decrease the incidence of cervical cancer.

## References

[1] Jemal A, Bray F, Center MM, Ferlay J, Ward E, Forman D Global cancer statistics. Cancer J Clin 2011;61(2):69–90. PMID: 10.3322/caac.20107 21296855

[2] Cancer in South Africa Report [homepage on the Internet]. 2009 [cited 2018 Sept 30]. p. 12–16. Available from: http://www.nioh.ac.za/assets/files/NCR_2009_FINAL.pdf.

[3] Bruni L, Albero G, Serrano B, et al ICO/IARC Information Centre on HPV and Cancer (HPV Information Centre) Human Papillomavirus and Related Diseases in South Africa. Summary Report 10 December 2018 Spain: HPV Information Centre.

[4] Moodley M Cervical cancer in Southern Africa: The challenges. S Afr J Gynaecol Oncol. 2009;1(1):11–13. 10.1080/20742835.2009.11441128

[5] Poole J, Patnick J Profile of cervical cancer in England incidence: Mortality and survival report Trent Cancer Registry. Sheffield: Trent Cancer Registry/NHS Cancer Screening Programmes; 2012.

[6] Hoffman A, Cooper D, Carrara H, et al Limited Pap screening associated with reduced risk of cervical cancer in South Africa. Int J Epidemiol. 2003;32:573–577. PMID: 10.1093/ije/dyg081 12913031

[7] Hoque M, Hoque E, Kader SB Evaluation of cervical cancer screening program at a rural community of South Africa. East Afr J Public Health. 2008;5(2):111–116. PMID:. 19024420

[8] Cervical Cancer Prevention and Control Policy, National Department of Health of South Africa [homepage on the Internet]. June 2017 [cited 2019 Apr 10]. Available from: file:///C:/Users/miche/Downloads/cervical%20cancer%20policy.pdf.

[9] World Health Organisation guidelines for screening and treatment of precancerous lesions for cervical cancer prevention [homepage on the Internet]. 2013 [cited 2018 Sept 30]. Available from: http://apps.who.int/iris/bitstream/10665/94830/1/9789241548694_eng.pdf.

[10] De Vuyst H, Ndirangu G, Moodley M, et al Prevalence of human papillomavirus in women with invasive cervical carcinoma by HIV status in Kenya and South Africa. Int J Cancer. 2012;131(4):949–955. PMID: 10.1002/ijc.26470 21960453

[11] National Department of Health of South Africa The 2013 National Antenatal Sentinel HIV Prevalence Survey South Africa [homepage on the Internet]. 2015 [cited 2018 Sept 30]. Available from: http://www.kznhealth.gov.za/data/The-2013-National-Antental-Sentinel-HIV-Prevalence-Survey-South-Africa.pdf.

[12] National Department of Health of South Africa, South Africa The national antenatal sentinel HIV and syphilis prevalence survey in South Africa [homepage on the Internet]. 2011 [cited 2018 Sept 30]. Available from: http://www.avert.org/south-africa-hiv-aids-statistics.htm#sthash.OajWHKrZ.dpuf.

[13] Van Bogaert LJ Age at diagnosis of preinvasive and invasive cervical Neoplasia in South Africa HIV-positive versus HIV-negative women. Int J Gynecol Cancer. 2011;21(2):363–366. PMID: 10.1097/IGC.0b013e3182094d78 21270617

[14] Terre Blanche M, Durrheim K, Painter D, editors Research in practice. Applied methods for the social sciences. Cape Town: University of Cape Town; 2006.

[15] Luesley D, Leeson S Guidelines for the NHS Cervical Screening Programme Second edition NHSCSP Publication No 20 May 2010 [homepage on the Internet]. [cited 2015 Sept 15]. Available from: http://www.cancerscreening.nhs.uk/cervical/publications/nhscsp20.pdf.

[16] Abdullah F, O’Rorke M, Murray L, Su TT Evaluation of a worksite cervical screening initiative to increase Pap smear uptake in Malaysia: A cluster randomized controlled trial. Biomed Res Int. 2013;2013:572126. PMID: 10.1155/2013/572126 24073411 PMC3773923

[17] Mingo A, Panozzo CA, DiAngi YT, et al Cervical cancer awareness and screening in Botswana. Int J Gynecol Cancer. 2012;22(4):638–644. PMID: 10.1098/IGC.0b013e318249470a 22367370 PMC4437542

